# Respiratory Syncytial Virus Reinfections in Children in Western Australia

**DOI:** 10.3390/v15122417

**Published:** 2023-12-13

**Authors:** David A. Foley, Cara A. Minney-Smith, Wei Hao Lee, Daniel B. Oakes, Briony Hazelton, Timothy J. Ford, Ushma Wadia, Chisha Sikazwe, Hannah C. Moore, Mark P. Nicol, Avram Levy, Christopher C. Blyth

**Affiliations:** 1 Department of Microbiology, PathWest Laboratory Medicine WA, Nedlands, WA 6009, Australiaavram.levy@health.wa.gov.au (A.L.); christopher.blyth@uwa.edu.au (C.C.B.); 2 Wesfarmers Centre of Vaccines and Infectious Diseases, Telethon Kids Institute, University of Western Australia, Perth, WA 6009, Australiahannah.moore@telethonkids.org.au (H.C.M.); mark.nicol@uwa.edu.au (M.P.N.); 3 School of Medicine, University of Western Australia, Perth, WA 6009, Australia; 4 Department of General Paediatrics, Perth Children’s Hospital, Nedlands, WA 6009, Australia; 5 Department of Infectious Diseases, Perth Children’s Hospital, Nedlands, WA 6009, Australia; 6 Department of General Paediatrics, Fiona Stanley Hospital, Murdoch, WA 6150, Australia; 7 Marshall Centre for Infectious Diseases, School of Biomedical Sciences, University of Western Australia, Perth, WA 6009, Australia; 8 School of Population Health, Curtin University, Perth, WA 6009, Australia; 9 Marshall Centre, Biomedical Sciences, University of Western Australia, Perth, WA 6009, Australia

**Keywords:** respiratory virus, respiratory syncytial virus, immunity, reinfection, children

## Abstract

Respiratory syncytial virus (RSV) reinfection in children is poorly understood. We examined the incidence, characteristics, and outcomes of hospital-attended RSV reinfections in children <16 years in Western Australia between 2012 and 2022. Individuals with repeat RSV detections ≥56 days apart were identified using laboratory data. The incidence of reinfection in the first five years of life was estimated using the total birth population from 2012 to 2017. Clinical data on a subset of reinfection episodes were obtained from two metropolitan pediatric centers. A total of 466 children with hospital-attended reinfections were identified. The median interval between RSV detections was 460 days (interquartile range: 324, 812), with a reinfection rate of 95 per 100,000 individuals (95% confidence interval: 82, 109). Reinfection was most common in children who experienced their first RSV detection <6 months of age. Predisposing factors were identified in 56% of children; children with predisposing factors were older at first and second detections, were more likely to be admitted, and had a longer length of stay. This study highlights the significant burden of hospital-attended RSV reinfections in children with and without predisposing factors. Expanded surveillance with in-depth clinical data is required to further characterize the impact of RSV reinfection.

## 1. Introduction

Respiratory syncytial virus (RSV) is a leading cause of acute lower respiratory tract infections in children [1]. RSV infection can range from mild upper respiratory tract infection to severe lower respiratory infection, with 3.6 million children receiving hospital-level care for RSV-associated acute lower respiratory infection in the first five years of life [2]. Children born prematurely or with significant comorbidities (e.g., congenital heart disease) are at risk of more severe disease [3]. By two years of age, almost all children have experienced their first RSV infection [4]. 

Immunity post infection is incomplete [5]. Consequently, RSV reinfection can occur throughout life [6]. Reinfection within the same RSV season is possible but infrequent [7]. The severity and frequency of reinfections in childhood are, in part, dependent on the immune response to the primary infection [6]. The duration and adequacy of protection post infection are age dependent, with younger age at first infection a risk factor for more severe disease [5,6]. However, age-dependent host-related factors, such as airway size, physiological reserve, and immunologic maturity, may have a more important role than immunological memory in determining the severity of subsequent infections [8]. Although severe RSV reinfection is reported to be uncommon [9], a comprehensive understanding of the rate, characteristics, and outcomes is lacking. Interest in this area has grown, yet reports have predominantly been confined to at-risk populations, small cohorts, or hospital coding data [7,10].

The development of single-dose long-acting monoclonal antibodies and intensified efforts in RSV vaccine development have changed the RSV prevention landscape [11]. Passive immunization through new long-lasting monoclonals or maternal vaccination is expected to be central in delaying and mitigating the severity of primary infection in children [12]. These strategies may also potentially modify the frequency and severity of reinfection. A better understanding of RSV reinfection will be crucial to informing these programs and identifying those at risk of more severe reinfection outcomes. We used RSV laboratory data from a state-wide pathology provider and hospital data from two pediatric centers to estimate the rate, characteristics, and outcomes of hospital-attended RSV reinfection.

## 2. Methods

### 2.1. Study Location and RSV Seasonality

Western Australia (WA) is a large, sparsely populated state, measuring over 2.6 million km^2^, with over 80% of the 2.8 million population residing in the Perth Metropolitan region. Temperate regions in the southern parts of WA experience a single winter RSV season, typically lasting approximately 20 weeks, with year-round activity observed in the tropical north [13]. SARS-CoV-2 related non-pharmaceutical interventions disrupted RSV seasonality in 2020, with an absent winter season and subsequent out-of-season summer surge [14,15]. Seasonality has slowly returned to the more predictable winter peak [16]. 

### 2.2. Data Sources

#### 2.2.1. Laboratory Data

Laboratory data were obtained from PathWest Laboratory Medicine, the state reference laboratory. This laboratory provides respiratory virus testing services to all public hospitals in Western Australia, selected community-based healthcare facilities, and primary care practices in regional areas. This laboratory uses various nucleic acid amplification assays, including commercial assays such as the GeneXpert Xpress Flu/RSV (Cepheid, Sunnyvale, CA, USA) and a laboratory-developed test to detect RSV [17]. Before 2018, children attending the state quaternary pediatric hospital were initially tested for RSV by direct immunofluorescence (IMAGEN, ThermoFisher Scientific, Melbourne, Australia), followed by real-time polymerase chain reaction if negative. Nucleic acid amplification assays were the preferred primary test at other sites before 2018. A specific polymerase chain reaction determined the RSV subtype for a portion of RSV-positive samples [10]. 

For this study, RSV testing and detection data were obtained from PathWest Laboratory Medicine for individuals aged <16 years at the time of testing between 1 January 2012 and 31 December 2022. The extracted data included the age at the time of infection, sex, collection site (e.g., location of hospital/emergency department), and the RSV subtype (A or B), if available.

#### 2.2.2. Clinical Data Subset 

Laboratory data were supplemented with clinical data obtained from the electronic medical records for the subset of children attending Perth Children’s Hospital (PCH) and Fiona Stanley Hospital (FSH), the two metropolitan centers with the state’s largest pediatric emergency department attendances. PCH, formerly Princess Margaret Hospital, relocated to its new site in 2018. This 298-bed hospital is the only specialized pediatric quaternary healthcare facility in Western Australia, with almost 70,000 emergency department presentations annually [18]. The level-four 20-bed pediatric department at Fiona Stanley Hospital (FSH) was established in 2015 to support the provision of pediatric services in the southern metropolitan region of Perth and has over 30,000 pediatric emergency department presentations per year [19]. 

The electronic discharge summary and laboratory results from children admitted to PCH and FSH were reviewed by two pediatric physicians (DAF, WHL). Admission data were unavailable for children admitted to other health facilities in the state. Clinical assessments and hospitalizations occurring within five days of RSV detections were identified. Data collected included whether the assessment was RSV-related, presence of predisposing conditions (as per definition in subsequent section), the need for intensive care unit (ICU) admission, respiratory and nutritional support, other investigations (e.g., blood test or chest X-ray), antibiotic administration, discharge diagnosis, and length of stay (LOS).

### 2.3. Definitions

#### 2.3.1. Persistent Detection

Persistent detection of RSV can occur post infection [20], especially in individuals with an impaired immune response [21]. We adopted a pragmatic approach to identify persistent detection. Detections were defined as persistent if they occurred <56 days following an initial detection. This interval was supported by local typing data where the shortest interval between infection with alternating RSV types was consistently greater than 70 days. To determine persistence ≥56 days, detections between 56 and 100 days apart and detections separated by >100 days in children who are immunocompromised were individually reviewed by a pediatric physician (DAF). Detections ≥56 days apart were defined as persistent in the presence of a combination of (i) an alternative diagnosis/etiology for presentation; (ii) repeat detection near the limit of detection of the test (e.g., cycle threshold >35, or discrepant result on the same sample when tested by a second molecular assay), or decreasing viral detection strength in multiple tests spanning ≥56 days; (iii) a predisposing condition that is associated with impaired immune response to infection (Table S1). Detections determined to be persistent were excluded from further analysis. 

#### 2.3.2. Predisposing Factors

Comorbid conditions known to reduce physiological reserve, alter the risk of infection severity, or modify management were characterized as predisposing conditions [3]. Individuals were determined to have a predisposing factor if such a condition was present at any hospital-attended RSV detection. Prematurity was defined as birth gestational age <37 weeks, with <32 weeks as very to extremely premature [22]. Significant congenital heart disease was defined as pulmonary hypertension, heart disease requiring diuretics, or uncorrected or palliated cyanotic heart disease [22]. Neurological and neuromuscular conditions (e.g., hypotonia, seizure disorders, moderate to severe cerebral palsy) and syndromes (e.g., trisomy-21) were grouped. Respiratory predisposing factors included chronic lung disease of prematurity, asthma (receiving at presentation or commenced on an inhaled corticosteroid), airway disease requiring tracheostomy, and conditions that predispose to recurrent lower respiratory tract infections (e.g., cystic fibrosis, bronchiectasis, or significant bronchomalacia). Children receiving chemotherapy with persistent agranulocytosis or hypo/asplenia were grouped and labeled as having immunodeficiency. Predisposing factor data were only available for children that attended PCH or FSH.

#### 2.3.3. Outcomes

Respiratory support included low-flow oxygen, humidified high-flow nasal oxygen, continuous positive airway pressure, and mechanical ventilation. Humidified high-flow nasal oxygen and continuous positive airway pressure were grouped and defined as pressure support. Nutritional support included nasogastric/orogastric placement or intravenous fluids. Clinical diagnosis was defined by site of infection and clinical phenotype in the electronic discharge summary, grouped into acute lower respiratory tract infection (ALRI), wheeze responsive to salbutamol (viral-induced wheeze, VIW), upper respiratory tract infection (URTI), and other. 

### 2.4. Statistical Analysis

Data analyses were predominantly descriptive and conducted using Stata/IC, V.11.2 statistical software (Stata Corp, College Station, TX, USA). χ2 was used to compare proportions between different groups, while the Mann–Whitney U test was employed to compare non-normally distributed continuous variables. An incidence rate was estimated using the number of hospital-attended RSV reinfection episodes in the first five years of life of children born between 2012 and 2017, divided by the number of children at risk (labeled the 2012–2017 Incidence rate sub-group), estimated from the Australian Bureau of Statistics [23]. Children transferred from or to another hospital or where the admission was not primarily related to RSV were excluded from the length-of-stay analysis. A Sankey figure was generated using SankeyMATIC [24]. 

## 3. Results

**Figure 1:**
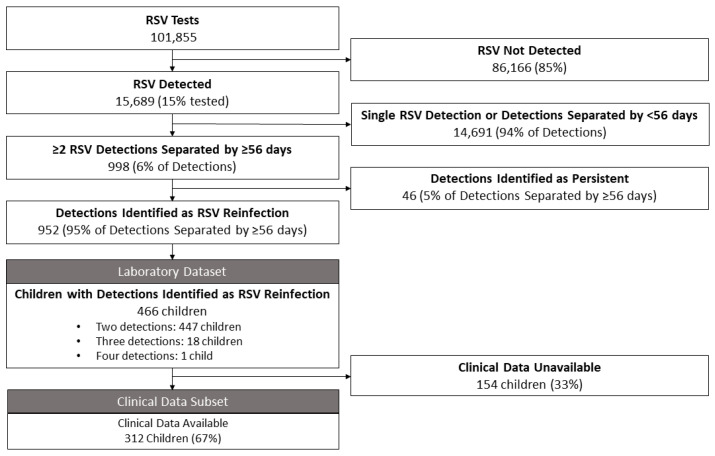
Flow diagram of children tested for RSV between 2012 and 2022 with RSV detection ≥56 days apart and subset with available clinical data.

**Figure 2:**
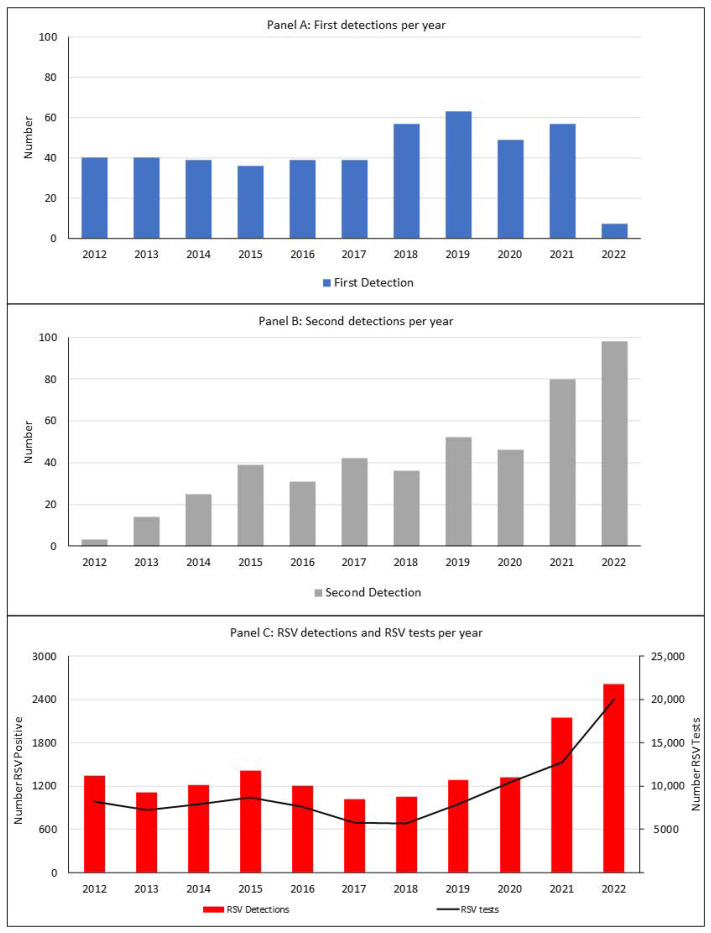
(**Panel A**) The number of first RSV detections per year of children with hospital-attended RSV reinfection. (**Panel B**) The number of second RSV detections per year of children with hospital-attended RSV reinfection. (**Panel C**) Total RSV detections and tests per year of all children <16 years in Western Australia. RSV, respiratory syncytial virus.

There were 101,855 tests conducted for RSV over the study period (Figure 1), with 15,689 detections (15.4% positive). RSV testing and detections were highest in 2021 and 2022 (Figure 2). There were 998 repeat detections separated by ≥56 days from 486 children were identified. Of these, 46 detections were classified as persistent detections (Table S1). A total of 466 children with hospital-attended RSV reinfection (952 detections) were included in this study, of which 58% were male, and 63% resided in the Perth Metropolitan region.

In those with reinfection, the frequency of primary detections increased in 2018 and 2019, decreasing in 2020, corresponding with the delayed RSV season and out-of-season resurgence. The lowest frequency of first detections in the reinfection group occurred in 2022, consistent with a limited opportunity for reinfection. Similarly, second RSV detections were lowest between 2012 and 2014, aligned with the cumulative number of children at risk of reinfection during that period. The number of second RSV detections was highest in 2022 (Figure 2).

Table 1 demonstrates the median age of those with hospital-attended reinfection and interval to subsequent RSV detection. The shortest interval to reinfection was in an infant born prematurely (34 weeks) with a first out-of-season infection at 41 days of life, followed by a second infection with a different RSV subtype 74 days later.

There were 3103 per 100,000 first detections (95% confidence interval [CI], 3028 to 3179) in the 2012–2017 Incidence Rate subgroup; of these, 3.1% had hospital-attended RSV reinfection (95% CI, 2.5–3.5%; 95 episodes per 100,000 individuals, 95% CI, 82 to 109). There was no statistically significant difference in the median interval between second and third RSV detection in all children and the 2012–2017 incidence rate subgroup. 

Hospital-attended RSV reinfection rate per person days, estimated using birth data for the region, was almost three times higher in children with infection in the first six months of life than all other groups (incidence rate ratio of 2.8, [95% CI 2.3 to 3.5] for <6 months compared with ≥6 months to <24 months at first detection). Children with the first infection ≥6 months to <24 months had a shorter interval to hospital-attended reinfection than those in the <6 months (*p* < 0.001) and ≥24 months age groups for the second detection (*p* < 0.001). There was no statistical difference for the interval to subsequent infection between <6 months and ≥24 months age groups. 

### 3.1. Subtyping Data 

Paired subtyping data (first and second event) were available for 156 hospital-attended reinfections, representing 33% of the total; of these, 58 (37%) were the same subtype and 98 (63%) alternating subtypes (*p* < 0.0001). There was no significant statistical difference for median age at onset or interval to subsequent detections between alternating and the same subtype (*p* > 0.05).

### 3.2. Clinical Data Subset

Clinical data were available for 312 (67%) children with hospital-attended reinfection. Of these, 56% (N = 175) were male, and 80% (N = 252) resided in the metropolitan region. A predisposing factor was identified in 56% (N = 175). The relative proportion of individuals with predisposing factors changed over the study, decreasing in 2020–2022 compared with previous years (43% vs. 63% in 2012–2019, *p* < 0.001). Prematurity (29%) was the most common predisposing factor identified, followed by respiratory conditions (24%) and neurology/neuromuscular conditions (17%) (Table 2).

**Figure 3:**
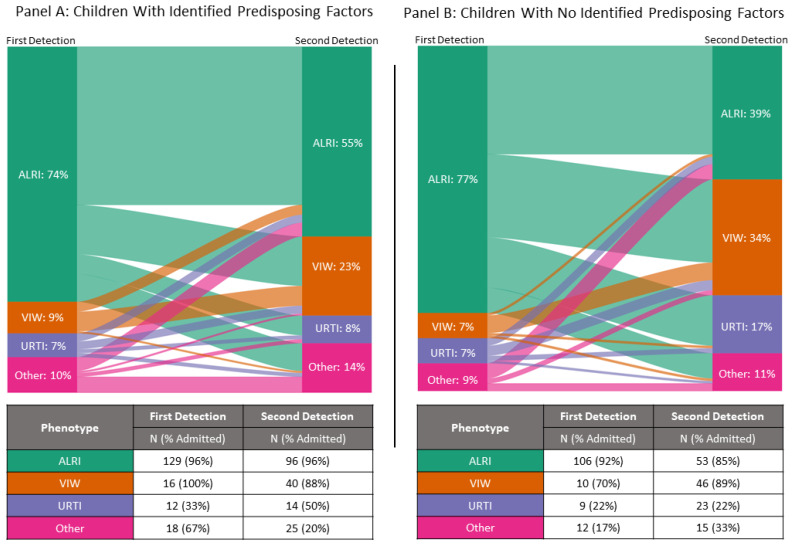
Sankey diagram of paired clinical phenotype at first and second detections in children with and without identified predisposing factors and the number and percentage of each phenotype admitted. ALRI, acute lower respiratory infection; URTI, upper respiratory tract infection; VIW, viral induced wheeze. “Other” includes febrile seizure, fever in at-risk individuals, and detections without clinical phenotype data.

Children with predisposing factors were older at first detection (*p* < 0.001), were more likely to be admitted (*p* = 0.03), and had a longer length of stay (*p* < 0.001) than those without identified predisposing factors (Table 3). ALRI was the most common admission type for children with and without identified predisposing factors (Figure 3). Among children without identified predisposing factors, the majority (N = 71, 52%) experienced their first RSV infection before six months of age. The most common age group for first infections in children with predisposing factors was ≥6 months to <24 months (N = 70, 40%). Despite a smaller proportion of children with identified predisposing factors having their first infection before six months (N = 51, 29%), this group had the highest hospital-attended reinfection rate.

Within the clinical dataset, the median interval to the second detection was 458 days (IQR, 325 to 812), with no significant difference between those with and without predisposing factors (*p* > 0.05). ALRI remained the most common RSV reinfection presentation type in children with predisposing factors, with the ALRI proportion decreasing in children without identified predisposing factors (39% vs. 55% in children with predisposing factors *p* = 0.005). Compared with the first detection, the proportion admitted was lower (*p* > 0.05), and the length of stay was shorter for children with (*p* < 0.001) and without identified predisposing factors (*p* < 0.001) at the second event.

There was minimal difference in the management of children with and without identified predisposing factors during their first RSV-related admission (Table S2). Children with predisposing factors were more likely to receive nutritional support, respiratory support, and antibiotics during the second (*p* < 0.002). Although the proportion receiving interventions was generally lower when comparing first and second RSV-related admissions in children with predisposing factors, respiratory support remained frequent (67%), antibiotic administration was higher (58%), and intensive care support remained common (11%).

## 4. Discussion

The findings of this study provide insight into the frequency and characteristics of hospital-attended RSV reinfection in children. We found a hospital-attended reinfection rate of 95 per 100,000 children in the first five years of life. Hospital-attended reinfections were more frequent in children with their first infection <6 months of age, irrespective of predisposing factor status. There was no observed difference between the median age at onset and interval to subsequent detections between alternating and the same RSV subtype. Children aged ≥6 months to <24 months at first detection had the shortest interval to subsequent hospital-attended RSV infection. Children with predisposing factors were older at first and second detections, were more likely to be admitted, and had a longer hospital stay. 

Hospital-attended RSV reinfection was observed in 3% of children. This number is almost ten times higher than the inpatient reinfection rate reported by Nduaguba et al. [7], approaching the outpatient-attended reinfection rate in their report of 34.4 per 1000 children with primary infection. Several factors may have contributed to this observed difference, including differing population sociodemographic characteristics, alternative healthcare delivery models, and the utilization of coding data to identify reinfection. Further, the accurate ascertainment of the burden of hospital-attended RSV reinfection also relies on respiratory virus testing practices; incomplete testing will contribute to the under-ascertainment of the true incidence of RSV reinfection. In the pre-SARS-CoV-2 period, the state quaternary pediatric hospital had a testing rate of 60% in children with respiratory-related admissions in 2019 [15]. Testing rates were lower in older children and those presenting with wheezing clinical phenotypes, potentially leading to the underdiagnosis of reinfection. The emergence of SARS-CoV-2 was associated with increased testing rates, especially among older children [15]. This period of expanded testing was not fully captured by the incidence calculation used in this report. However, this shift in practice likely contributed significantly to the observed rise in the reinfection numbers in later years of the study, particularly among children without identified predisposing factors. If maintained, the expanded testing practices associated with SARS-CoV-2 will provide a more accurate understanding of the burden of hospital-attended RSV reinfection. 

Although hospital-attended RSV reinfection is relatively infrequent, these children, especially those with predisposing factors, received a high rate of medical intervention, including hospitalization, respiratory and nutritional support, and antibiotics. Interestingly, a large portion of children with reinfection had infections with alternating RSV types. This finding suggests that infection with a different RSV subtype may be more severe than sequential infection with the same type. However, subtyping data were only available for a small proportion of detections and several potential confounders exist, including variations from season to season in the prevalent subtype. 

Hospital-attended reinfection was more common in children with their first RSV infection before six months of age, irrespective of the presence or absence of predisposing factors. This increased rate may be partly attributed to an inadequate or potentially inappropriate immune response following a first infection [4]. However, age-related physiological and immunological differences are expected to be central in determining disease severity, at both the first and second infection [8]. 

Preventing RSV infection in children younger than six months may have multiple benefits, including reducing the risk of subsequent hospital-attended reinfection. Utilizing long-acting monoclonal antibodies in this age group or maternal vaccination could be crucial in achieving this goal. These antibodies may delay or mitigate the severity of the initial RSV infection. Infection in older children is typically milder. Further, older age at first infection may provide more substantial immune protection [6], in addition to the important size-related physiological changes with age, lowering the risk of subsequent hospital-attended reinfection. This reduction may enhance the potential cost-effectiveness of broadly implementing long-acting monoclonals in children. 

The consistent interval between the first and second RSV infections, regardless of the presence or absence of predisposing factors, suggests a comparable pattern of susceptibility and exposure. Similarly, the absence of a difference in interval between alternating and same RSV subtypes also suggests that, although the severity of infection may differ, this interval depends more on exposure than differing susceptibility profiles. This finding is further supported by the shorter interval observed in children with the first infection between 6 months and 24 months compared with the other age cohorts. Although this difference may be linked to an overly robust immune response to subsequent infections, exposure is expected to be the critical driver. Children in this age range are more likely to be in close contact with other individuals, such as in early learning centers, increasing their exposure to RSV. 

It is unsurprising that prematurity and respiratory and neurological/neuromuscular conditions were the most common predisposing factors. These conditions have been identified as risk factors for RSV lower respiratory tract infection [25,26]. Children with Trisomy 21 were over-represented, consistent with a clustering of risk factors for more severe infection in this group. These findings emphasize the vulnerability of these populations to more severe RSV disease and the need for targeted interventions and preventive measures. 

The older age at onset for children with predisposing factors is likely linked to prolonged hospitalization at the start of life, protective behaviors, and the use of palivizumab in this high-risk population by the local pediatric centers [27]. Despite being older at the second RSV detection, children with predisposing factors were more likely to present with ALRI, be admitted, and have a longer hospital stay. Additionally, these children received significantly higher levels of gastrointestinal and respiratory support, consistent with a more severe phenotype. These findings highlight the importance of predisposing conditions in determining the severity and clinical outcomes. Children with significant risk factors may benefit from continued access to long-acting monoclonal RSV immuno-prophylaxis at an older age.

### Limitations

The absence of data from private pathology providers and private pediatric inpatient facilities and limited community-level testing in this study may have led to an underestimation of both primary and subsequent RSV infections. The pragmatic approach used to differentiate reinfection from persistence may have incorrectly excluded individuals with reinfection. The estimation of reinfection incidence did not consider population dynamics and the geographical locations of birth. Additionally, clinical data were only obtained from two metropolitan hospitals, including a quaternary center, which may result in the over-representation of children with risk factors and severe disease in the clinical dataset, thus limiting the generalizability of the findings to other healthcare settings. Clinical data were also limited to electronic discharge summaries, which may have led to an underestimation of the interventions provided. The impact of co-infection with other respiratory viruses such as rhinovirus and adenovirus, was not assessed due to limited testing data. Consequently, unrecognized respiratory virus coinfection may have contributed to an overestimation of both hospital-attended RSV reinfection rates and associated severity.

## 5. Conclusions

Hospital-attended RSV reinfection is an important but underexplored condition affecting children with and without predisposing factors. Our study highlights the need for further research and surveillance to understand reinfection dynamics and inform preventive strategies, including RSV immunizations. Expanded community-level surveillance is required to fully characterize the frequency and impact of RSV reinfection in children. 

## Figures and Tables

**Table 1:** The median age and interval to subsequent detection in months by all identified individuals in the study period and by age at first infection cohorts. IQR, interquartile range; N, number.

Group	N	Median Age (IQR)	Comparison With Reinfection Group
All RSV First Detections	15,689	12 months (4–26)	*p* = 0.5
**Reinfection Group**	**N**	**Median Age (IQR)**	**Median Interval (IQR) to Subsequent Detection**
First Detection	466	9 months (3.6 to 20)	460 days (324 to 812)
Second Detection	466	29 months (18 to 50)	447 days (369 to 852)
Third Detection	19	52 months (34 to 73)	530 days
Fourth Detection	1	48 months	-
**Age Group at 1st Detection**	**N**	**Median Age (IQR)**	**Median Interval (IQR) to Subsequent Detection**
<6 months	178	3 months (2 to 4)	470 days (333 to 877)
≥6 months to <24 months	190	12 months (8 to 17)	393 days (282 to 707)
≥24 months	98	37 months (30 to 50)	644 days (368 to 1047)

**Table 2:** Children identified with predisposing factors for RSV infection by number of detections and by predisposing factor subtype. T21, trisomy 21. LRTI, lower respiratory tract infection.

Number	% with Predisposing Factors (N)
Two detections	54 (159)
Three detections	100 (15)
Four detections	100 (1)
**Predisposing Factor**	**% (N)**
Any risk factor	56 (175)
Prematurity (<37 weeks)	29 (89)
<32-week gestation	12 (38)
Respiratory Condition	24 (76)
Recurrent LRTIs	12 (37)
Chronic Lung Disease of Prematurity	10 (31)
Asthma	5 (15)
Tracheostomy in situ	3 (8)
Significant Heart Disease	5 (16)
Neurology/neuromuscular/syndromes	17 (53)
T21	2 (8)
Immunodeficiency	4 (14)

**Table 3:** Median age, percentage admitted, and length of stay for first and second detections by all children, children with identified predisposing factors, and children with no identified predisposing factors. *p*-values were calculated by comparing the subgroups using χ2.

	All	Predisposing Factors	No Predisposing Factors	*p*-Value
Median Age at First Detection, Months (IQR)	9 months	12.8 months	5.5 months	<0.0001
	(3.5–21.3)	(5.2 to 27.7)	(2.3 to 14.4)	
% First Detection Admitted (N)	85%	89%	80%	0.03
	(265)	(156)	(109)	
Length of Stay, Days (IQR)	3.2 days	4 days	2.6 days	<0.0001
	(1.7 to 6)	(2.1 to 7.2)	(1 to 4.7)	
Median Interval to Second Detection, Days (IQR)	458 days	462 days	456 days	>0.05
	(325 to 812)	(335 to 951)	(314 to 692)	
Median Age at Second detection, Years (IQR)	2.3 years	3 years	1.9 years	<0.0001
	(1.5 to 4.3)	(1.8 to 4.8)	(1.3 to 3.3)	
% Second Detection Admitted (N)	79%	83%	70%	0.007
	(242)	(146)	(96)	
Length of Stay for Second Detection, Days (IQR)	1.8 days	2.5 days	0.9 days	<0.0001
	(0.8 to 3.4)	(1.4 to 4.7)	(0.6 to 1.8)	

## Data Availability

The data presented in this study are available on request from the corresponding author.

## Supplementary Materials

The following supporting information can be downloaded at https://www.mdpi.com/article/10.3390/v15122417/s1, Table S1: The detection profile and rationale for detections identified as persistent; Table S2: The inpatient management of the first and second detection.
