# Supplementary material for: Respiratory Syncytial Virus Reinfections in Children in Western Australia
Source: Viruses. 2023 Dec 13;15(12):2417. doi: 10.3390/v15122417 (PMC10747877; doi:10.3390/v15122417)
Supplement: Supplementary file 1 [file viruses-15-02417-s001.zip › viruses-2749809-supplementary.pdf]

viruses

Article

Respiratory Syncytial Virus Reinfections in Children in Western Australia

David A. Foley [1,2,3,*], Cara A. Minney-Smith [1], Wei Hao Lee [3,4], Daniel B. Oakes [2], Briony Hazelton [1,5], Timothy J. Ford [3,5], Ushma Wadia [2,5,6], Chisha Sikazwe [1,7], Hannah C. Moore [2,8], Mark P. Nicol [2,9], Avram Levy [1,7] and Christopher C. Blyth [1,2,3,5]

1 Department of Microbiology, PathWest Laboratory Medicine WA, Nedlands, WA 6009, Australia; cara.minney-smith@health.wa.gov.au (C.A.M.-S.); briony.hazelton@health.wa.gov.au (B.H.); chisha.sikazwe@health.wa.gov.au (C.S.); avram.levy@health.wa.gov.au (A.L.); christopher.blyth@uwa.edu.au (C.C.B.)
2 Wesfarmers Centre of Vaccines and Infectious Diseases, Telethon Kids Institute, University of Western Australia, Perth, WA 6009, Australia; daniel.oakes@telethonkids.org.au (D.B.O.); ushma.wadia@health.wa.gov.au (U.W.); hannah.moore@telethonkids.org.au (H.C.M.); mark.nicol@uwa.edu.au (M.P.N.)
3 School of Medicine, University of Western Australia, Perth, WA 6009, Australia; weihao.lee@health.wa.gov.au (W.H.L.); tim.ford@health.wa.gov.au (T.J.F.)
4 Department of General Paediatrics, Perth Children's Hospital, Nedlands, WA 6009, Australia
5 Department of Infectious Diseases, Perth Children's Hospital, Nedlands, WA 6009, Australia
6 Department of General Paediatrics, Fiona Stanley Hospital, Murdoch, WA 6150, Australia
7 Marshall Centre for Infectious Diseases, School of Biomedical Sciences, University of Western Australia, Perth, WA 6009, Australia
8 School of Population Health, Curtin University, Perth, WA 6009, Australia
9 Marshall Centre, Biomedical Sciences, University of Western Australia, Perth, WA 6009, Australia
* Correspondence: david.foley@telethonkids.org.au

Table S1. The detection profile and rationale of detections identified as persistent.

ID	Detections excluded	Interval (days)	Age-Group at first detection	Rationale
1	1	74	1-4 yrs	Late detection for second detection (>35) with discrepant results on the second assay.
2	1	76	1-4 yrs	Hyperinsulinemia, congenital central hypoventilation syndrome with tracheostomy; late detection (>35) for second with discrepant detections results on additional testing. 2nd detection occurred when presenting with constipation.
3	1	76	1-4 yrs	Extreme prematurity, inhaled fluticasone; on hydrocortisone replacement therapy; interval positive results, same RSV type with late second detection. Other viral aetiology identified for respiratory presentation (parainfluenza).
4	1	96	<1 yr	Multiple complex congenital anomalies with immunodeficiency and respiratory muscular insufficiency. Same RSV type (A) with late detection (35) for this test, with other discrepant results.
5	1	105	<1 yr	Syndrome with immunodeficiency and agenesis of the corpus callosum. Late detection for second with the same type (B). Non-respiratory presentation for the second detection.
6	1	126	5-15 yrs	Cystic fibrosis on oral steroids for allergic bronchopulmonary aspergillosis and specific antibody deficiency. Multiple interval detections.

7	2	56	<1 yr	Same RSV type (type B), late detection (>35) for second; presentation for second detection with gastrointestinal upset
8	2	57	<1 yr	Tracheomalacia. Testing for the second detection was undertaken as part of a planned bronchoscopy for tracheomalacia. Late detection for the second detection (>35) and discrepant results on the alternative test.
9	2	57	<1 yr	Late detection on second detection (>35). Non-respiratory presentation for the second test.
10	2	57	5-15 yrs	Profoundly immunocompromised with lymphoproliferative syndrome with multiple detections in the intervening period. No new respiratory symptoms
11	2	59	1-4 yrs	Extreme prematurity. Second detection (>35) with discrepant results on the second test. An alternative virus was detected that was attributed to presentation (adenovirus).
12	2	60	1-4 yrs	Hepatoblastoma on treatment; multiple interval detections with declining detection strength.
13	2	62	1-4 yrs	Prematurity. Late detection (>35) for the second detection with the same type detected (A).
14	2	63	<1 yr	Prematurity. Late detection for the second (>35) with an alternative virus detected that was attributed to presentation (adenovirus).
15	2	63	1-4 yrs	Renal cell sarcoma on treatment with multiple detections over the intervening period.
16	2	64	<1 yr	Extreme prematurity: Second detection at the detection limit with a discrepant second test. Other respiratory virus detected (parainfluenza)
17	2	64	<1 yr	Lympho-vascular malformation of tongue; late second detection (>35). 2nd presentation for additional imaging; no respiratory symptoms
18	2	66	<1 yr	Trisomy 21, severe pulmonary hypertension, poor weight gain. Multiple interval detections with declining strength; same RSV type (B). No new clinical symptoms with detection at 66 days
19	2	71	5-15 yrs	Acute lymphoblastic leukaemia; same RSV type (A) with multiple interval detections.
20	2	75	<1 yr	Multiple interval detections: late detection (>35) on one assay at day 75, with discrepant results on repeat testing the next day. Another new respiratory virus was detected (human metapneumovirus).
21	2	75	1-4 yrs	Viral-induced wheeze with two five-day courses of oral prednisolone in between—interval positives with late second detection of same RSV type (B).
22	2	77	<1yr	Investigated for immunodeficiency for recurrent infections, failure to thrive, and recurrent unexplained hypoglycaemia. Multiple interval detections with declining detection strength. A new virus was detected (adenovirus); the second presentation was associated with fever.
23	2	77	1-4 yrs	Acute lymphocytic leukaemia; same RSV type, declining detection strength, no new symptoms.
24	2	78	1-4 yrs	On fluticasone, asthma; mannose-binding lectin deficiency. Treated with oral prednisolone at first presentation for five days. Admitted with fever; adenovirus LRTI. Late detection of RSV (>35).
25	2	86	<1 yr	Extreme prematurity, late detection (>35) for second, with other interval detection at 46 days; same RSV type; no respiratory symptoms at day 86.

				alternative aetiology identified for presentation- gastrointestinal upset with cryptosporidium detected.
26	2	94	<1 yr	Prematurity. Late detection for second detection (>35) with discrepant results on the second assay.

Table S2. The inpatient management of the first and second detection. P-values were calculated by comparing the subgroups using χ2; -, p>0.05. GI, gastrointestinal.

**First Detection**	**All**	**Predisposing Factor**	**No Predisposing Factor**	**p-value**
**Management**	237	139	98	
	% (N)	% (N)	% (N)	
Nutritional Support (Any)	62% (147)	64% (89)	59% (58)	-
• *Intravenous fluids*	31% (73)	34% (47)	26% (26)	-
Respiratory support (any)	74% (178)	76% (107)	72% (71)	-
• *Low flow*	44% (104)	41% (58)	46% (46)	-
• *Pressure support*	27% (64)	31% (43)	21% (21)	-
• *Mechanical ventilation*	4% (10)	4% (6)	4% (4)	-
Antibiotics	46% (111)	52% (73)	38% (38)	-
Chest x-ray	51% (121)	55% (77)	44% (44)	-
Phlebotomy	56% (134)	61% (86)	48% (48)	0.048
Intensive Care	14% (33)	16% (23)	10% (10)	-

**Second Detection**	**All**	**Predisposing Factor**	**No Predisposing Factor**	**p-value**
**Management**	219	128	91	
Nutritional Support (Any)	36% (79)	45% (57)	24% (22)	0.002
• *Intravenous fluids*	25% (54)	32% (41)	14% (13)	0.002
Respiratory support (any)	57% (125)	67% (86)	43% (39)	0.0004
• *Low flow*	34% (75)	38% (48)	30% (27)	-
• *Pressure support*	22% (48)	29% (37)	12% (11)	0.003
• *Mechanical ventilation*	1% (2)	1% (1)	1% (1)	-
Antibiotics	47% (104)	58% (74)	33% (30)	0.001
Chest x-ray	48% (106)	54% (69)	41% (37)	-
Phlebotomy	51% (111)	59% (76)	38% (35)	0.002
Intensive Care	9% (20)	11% (14)	7% (6)	-
